# Impact of type 2 diabetes on life expectancy and role of kidney disease among inpatients with heart failure in Switzerland: an ambispective cohort study

**DOI:** 10.1186/s12933-023-01903-7

**Published:** 2023-07-12

**Authors:** Dante Salvador, Arjola Bano, Faina Wehrli, Valentina Gonzalez-Jaramillo, Markus Laimer, Lukas Hunziker, Taulant Muka

**Affiliations:** 1 Institute of Social and Preventive Medicine, University of Bern, Bern, Switzerland; 2 Department of Cardiology, Inselspital, Bern University Hospital, Bern, Switzerland; 3 Graduate School for Health Sciences, University of Bern, Bern, Switzerland; 4 Department of Diabetes, Endocrinology, Nutritional Medicine, and Metabolism, Inselspital, University of Bern, Bern, Switzerland; 5 Epistudia, Bern, Switzerland

**Keywords:** Type 2 diabetes, Heart failure, Chronic kidney disease, Cardiovascular prevention

## Abstract

**Background:**

Type 2 diabetes (T2D) is expected to worsen the prognosis of inpatients with heart failure (HF) but the evidence from observational studies is inconsistent. We aimed to compare mortality outcomes and life expectancy among inpatients with HF with or without T2D and explored whether chronic kidney disease (CKD) influenced these associations.

**Methods:**

We collected hospital and civil registry records of consecutive inpatients from a tertiary hospital in Switzerland with a diagnosis of HF from the year 2015 to 2019. We evaluated the association of T2D with mortality risk using Cox regression and adjusted for confounders.

**Results:**

Our final cohort consisted of 10,532 patients with HF of whom 27% had T2D. The median age (interquartile range [IQR]) was 75 [68 to 82] and 78 [68 to 86] for the diabetes and non-diabetes groups, respectively. Over a median follow-up [IQR] of 4.5 years [3.3 to 5.6], 5,347 (51%) of patients died. T2D patients had higher risk of all-cause mortality (hazard ratio [HR] 1.21, 95% confidence interval [CI] 1.14 to 1.29). Compared to control (i.e. no T2D nor CKD), average life expectancy (95% CI) among T2D patients, CKD, or both was shorter by 5.4 months (95% CI 1.1 to 9.7), 9.0 months (95% CI 8.4 to 9.6), or 14.8 months (95% CI 12.4 to 17.2), respectively. No difference by sex or ejection fraction category was observed.

**Conclusions:**

T2D is associated with a significantly higher risk of all-cause mortality and shorter life expectancy compared to those without among middle-aged and elderly inpatients with HF; presence of CKD may further increase these risks.

**Supplementary Information:**

The online version contains supplementary material available at 10.1186/s12933-023-01903-7.

## Introduction

Global trends on the burden of diabetes and heart failure (HF) continue to increase, thus highlighting the need for more effective preventive strategies [1, 2]. Type 2 diabetes (T2D) and HF individually confer considerable burden that are multiplied when these diseases co-exist, further decreasing patient’s quality of life and increasing healthcare costs [1]. In Switzerland, 500,000 (6%) of the population is estimated to have T2D and about 30–40% of them have chronic kidney disease (CKD) [3]. Meanwhile, cardiovascular diseases are main cause of mortality accounting for 27% of all deaths in 2020 [4], likely preceded by heart failure.

Hospitalised patients with HF represent advanced stages of disease with poor prognosis: high risk of in-hospital mortality from 4 to 7%, rehospitalisation from 25 to 30%, and mortality from 7 to 11%, with shorter life expectancy [2, 5]. While T2D is expected to worsen the prognosis of patients with HF, the evidence from observational studies has been inconsistent, with some studies showing adverse impact [6, 7], and some other studies reporting no impact on mortality [8, 9]. Moreover, these studies have mostly described short- and intermediate-term outcomes (i.e. up to 1-year) [6–9]. Meanwhile, CKD is prevalent among patients with T2D, and HF patients with CKD show higher risks for mortality than those without CKD [10]. Whether co-existence of both T2D and CKD in inpatients with HF would pose a higher burden on long-term mortality and life expectancy is not yet fully explored.

Studies that have explored the association between diabetes and mortality in HF patients did not estimate life expectancy of inpatients with HF, nor compare life expectancy between those with or without diabetes [11]. Previous studies limited their findings to hazard ratio estimates (instead of absolute risk estimates), that limits bed-side interpretability [7, 12, 13]. Similarly, some studies adjusted for presence of CKD, and but did not estimate the added mortality risk of CKD among inpatients with HF [11–13].

Therefore, by using data of inpatients with HF at the largest tertiary cardiovascular referral hospital in Switzerland, we compared mortality outcomes among patients with or without T2D and estimated the differences in life expectancy between groups. Additionally, we explored whether co-existence of T2D and CKD was associated with worse prognosis.

## Methods

### Study design, setting, and participants, and data collection

All patients with heart failure who were admitted at Bern University Hospital (Inselspital), Switzerland—a large tertiary cardiology center—between January 2015 and December 2019, were followed-up for the present study. Participants were excluded if they are 40 years old and younger, had type 1 diabetes, or developed diabetes after hospitalisation. The clinical data warehouse at the Inselspital contains administrative and medical data of all patients from any department including the Department of Cardiology. Information about diagnoses were obtained based on the International Statistical Classification of Diseases and Related Health Problems 10th version (ICD-10). In this study, we identified eligible participants based on relevant ICD-10 codes for heart failure (I50; I11.0; I09.81; I13.0; I13.2). Among other data, demographic and clinical characteristics, information on hospitalizations, comorbidities, implantable cardiac devices, heart failure and antidiabetic medications, laboratory data, and survival status were obtained. Demographic and clinical characteristics included age, sex, body mass index (BMI) and left ventricular ejection fraction (EF) measured using ultrasound. Patients were categorized as having heart failure with preserved ejection fraction (HFpEF, EF > 40) or reduced ejection fraction (HFrEF, EF ≤ 40%). Comorbidities included were T2D, CKD, hypertension, and atherosclerotic cardiovascular disease (ASCVD, defined as having at least one of the following: coronary artery disease, history of myocardial infarction, angina, and transient ischemic attack), and chronic obstructive pulmonary disease (COPD). Medication information was obtained using Anatomical Therapeutic Chemical Classification System (ATC) codes. Survival status was assessed by linking with the national mortality record. This study followed the Strengthening the Reporting of Observational Studies in Epidemiology (STROBE) guidelines [14].

### Statistical analysis

Data were summarized as mean (standard deviation, SD) or median [interquartile range] for continuous variables and as counts (proportions in %) for categorical variables.

Survival by T2D status and by sex for the overall inpatient population was presented using Kaplan–Meier (KM) curves. We estimated the association of T2D status (yes vs no) with the risk of all-cause mortality using Cox proportional hazards regression. In Model 1, we adjusted for age and sex (in overall analysis). In Model 2, we additionally adjusted for CKD, ASCVD, EF category, hypertension, atrial fibrillation, COPD, and dyslipidemia. In Model 3, we included interaction terms between T2D status, CKD status, and EF category. We tested for proportionality of hazards (PH) assumption using p > 0.05 as cut-off for Schoenfeld test and plotting Schoenfeld residuals against follow-up time. When PH assumption was not met, we stratified our analyses by time period: < 30 days (Period 1), 30 days to 1 year (Period 2), 1 to 2 years (Period 3), and > 2 years (Period 4), and reported hazard ratios (HRs) per period. Furthermore, we estimated whether the association between T2D and mortality differed by sex, EF category, or presence of CKD; for the last analysis we compared four patient groups (i) without T2D nor CKD (T2D-/CKD-); (ii) without T2D, with CKD (T2D-/CKD +); (iii) with T2D, without CKD (T2D + /CKD-); and (iv) with T2D and CKD (T2D + /CKD +). We compared life expectancy (mean survival in years) between (i) patients with and without T2D and (ii) T2D and CKD groups, by fitting a survival curve using a non-parametric step function [15]. We used bootstrapping with 1000 resamples to estimate the confidence intervals of the marginal changes in life expectancy associated with T2D (± CKD). Statistical analyses were performed using RStudio v.4.0.5 and packages: survival, survminer, and coxed [15].

### Sensitivity analysis

We performed sensitivity analysis by i) adjusting for, or ii) excluding patients who have died during that episode of hospital admission, herein defined as in-hospital mortality.

## Results

### Baseline characteristics

**Fig. 1:**
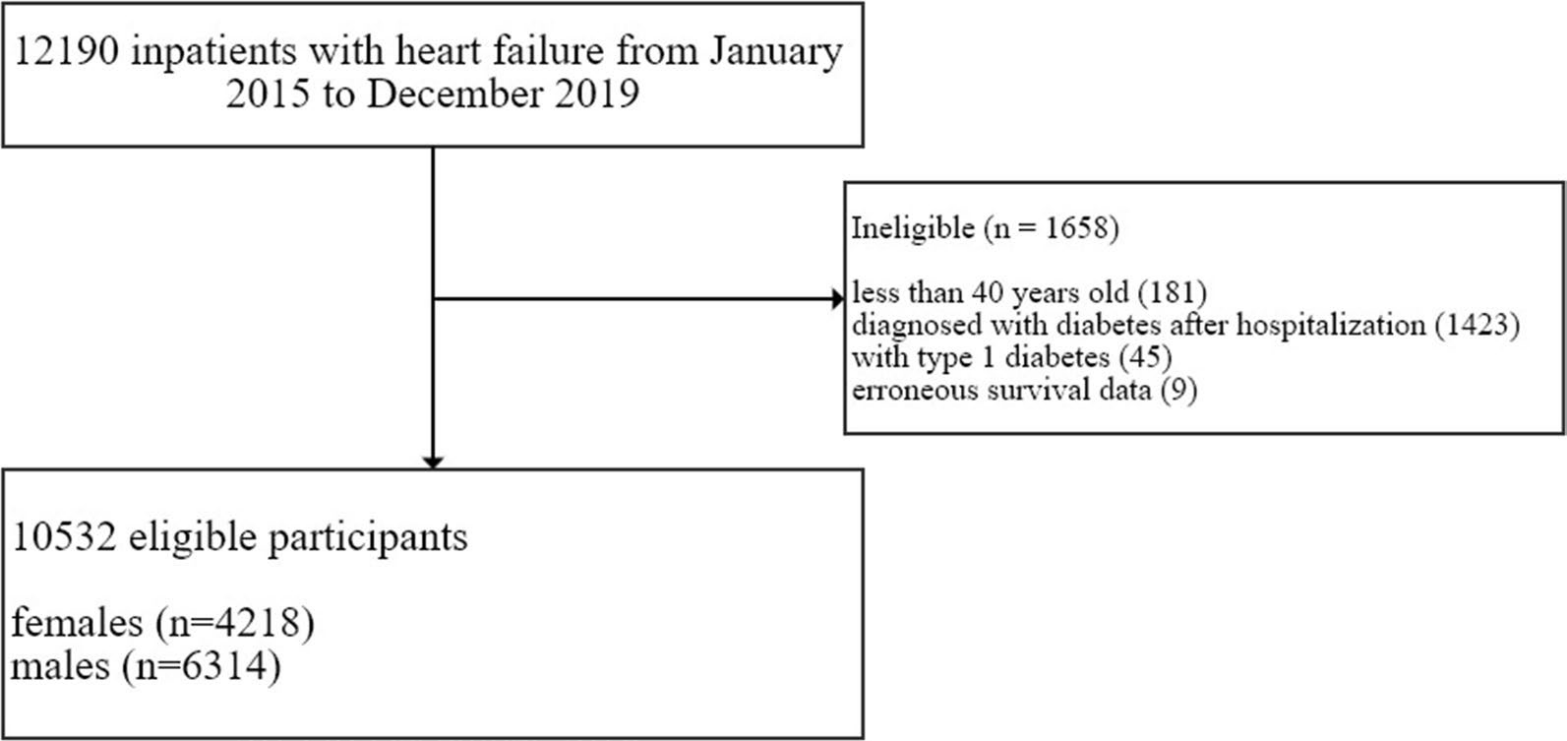
Flowchart of patient participation and eligibility. *erroneous survival data means their hospital admission date was after the registered date of death

A total of 12,190 inpatients with an ICD-10 diagnosis of HF (I50; I11.0; I09.81; I13.0; I13.2) from January 2015 to December 2019 were included in this study. Of these patients, 1658 were excluded because they did not meet eligibility criteria: developed T2D after hospitalization (n = 1423), < 40 years old (n = 181), with type 1 diabetes (n = 45), or erroneous civil registry data (n = 9). Our eligible study population was 10,532 patients with 4218 females (40%, median age = 81) and 6314 males (60%, median age = 74) (Fig. 1). Of these, 64% of patients had a BMI measurement and were included for our models adjusted further for BMI. T2D was prevalent in 2804 (27%) of the patients, with higher prevalence in males (29% vs 23% in females) (Table 1 and Additional file 1: Table S1). Among the most prevalent risk factors were hypertension (71% in T2D group vs 49% in control group), CKD (58% in T2D group vs 36% in control group), ASCVD (49% in T2D group vs 40% in control group), and atrial fibrillation (38% in T2D group vs 30% in control group). EF was similar between diabetes groups (median EF = 45%), with a similar prevalence of HFpEF and HFrEF at 58% and 42%, respectively. The median follow-up time [interquartile range] was 4.5 [3.3, 5.6] years, with 5347 (51%) deaths observed which were more prevalent in those with T2D (56% vs. 49%). Table 1 summarizes the population characteristics by T2D status.

**Table 1:** Baseline characteristics of inpatients with heart failure at Inselspital, 2015–2019

	No diabetes	Type 2 diabetes
n (%)	7728 (73%)	2804 (27%)
Sex (% males)	4468 (58%)	1846 (66%)
Age in years, median [IQR]	77.8 [67.6, 85.5]	75.2 [67.5, 82.0]
BMI^a^ in kg/m^2^, median [IQR]	25.6 [22.6, 29.1]	27.5 [24.2, 31.3]
Medical history		
Chronic kidney disease	2766 (36%)	1636 (58%)
ASCVD	3066 (40%)	1384 (49%)
Hypertension	3817 (49%)	1980 (71%)
Atrial fibrillation	2306 (30%)	1053 (38%)
Dyslipidaemia	108 (1%)	106 (4%)
COPD	726 (8%)	250 (14%)
Medications		
Insulin		2263 (81%)
Oral antidiabetic medications		1506 (54%)
Antithrombotic medications	6987 (90%)	2755 (98%)
Digoxins and nitrates	1851 (24%)	1049 (37%)
Diuretics	6075 (79%)	2658 (95%)
Beta-blockers	5779 (75%)	2356 (84%)
RAAS inhibitors^b^	5913 (77%)	2451 (87%)
Ejection fraction, % median [IQR]	45 [32, 60]	45 [35, 60]
HFpEF %	4512 (58%)	1636 (58%)
HFrEF %	3216 (42%)	1168 (42%)
Deaths	3770 (49%)	1577 (56%)

^a^ summary estimates based on non-missing values (only 6,740 or 64% of patients have had BMI measurements)

^b^ includes ACEi, aliskiren, ARB, ARNI

*ACEi* angiotensin-converting enzyme inhibitor, *ARB* angiotensin receptor blocker, *ARNI* angiotensin receptor-neprilysin inhibitor, *ASCVD* atherosclerotic cardiovascular disease, *BMI* body mass index; *COPD* chronic obstructive pulmonary disease, *RAAS* renin–angiotensin–aldosterone system

### Type 2 diabetes and mortality risk, and role of sex, ejection fraction, and chronic kidney disease

**Fig. 2:**
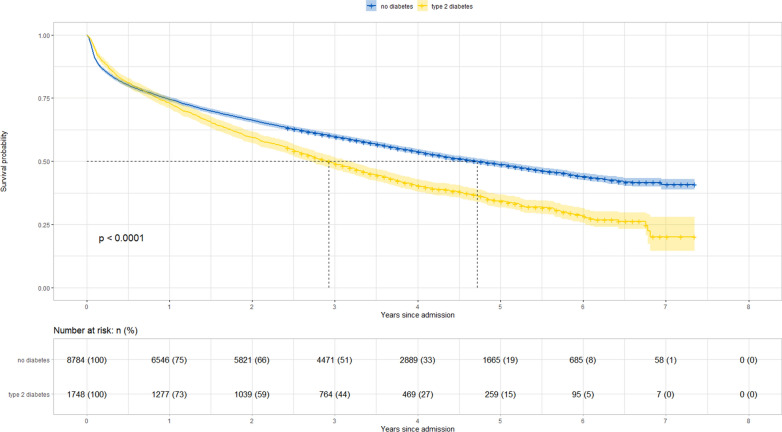
Survival curve of inpatients with heart failure, according to T2D status

**Fig. 3:**
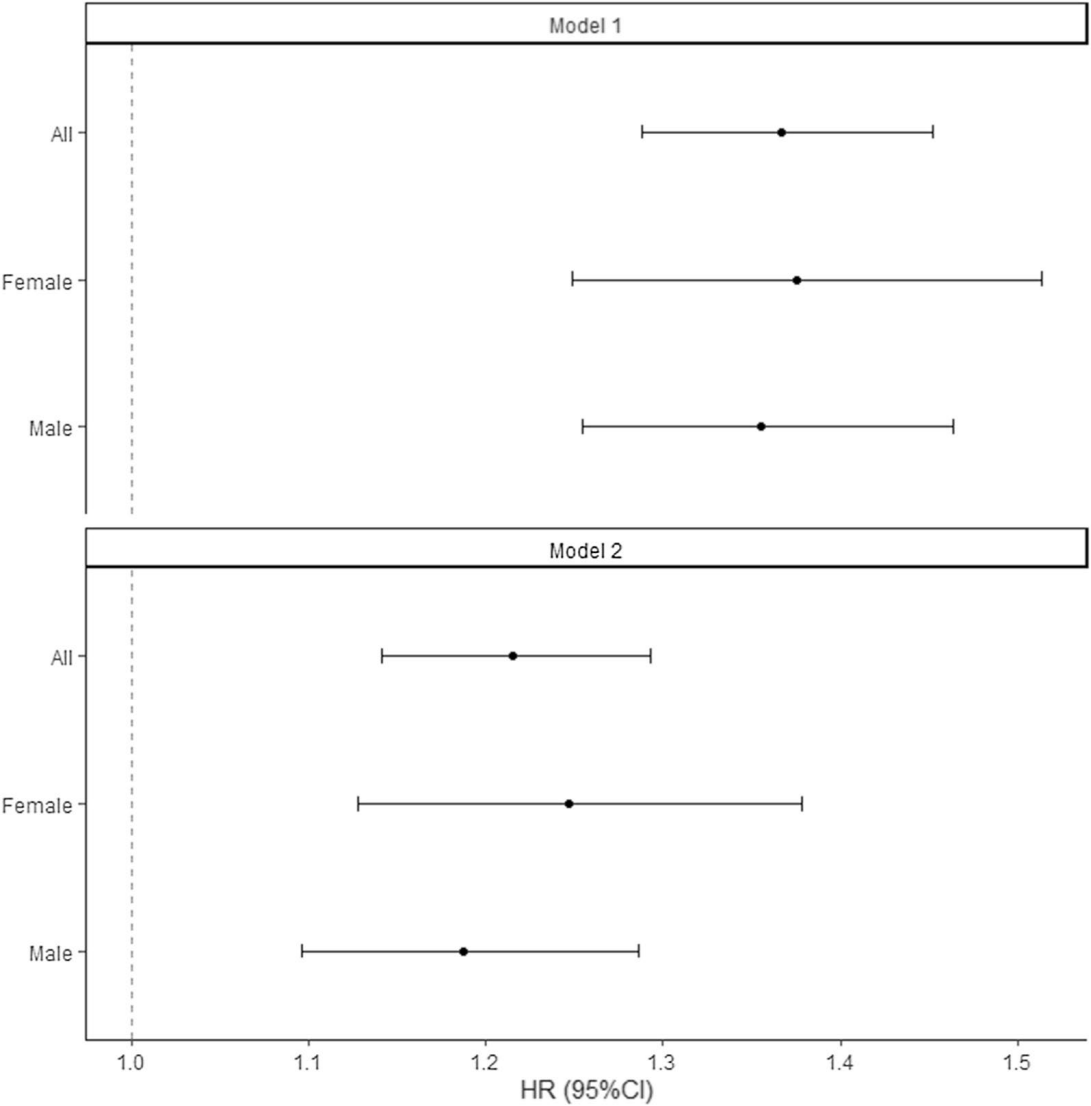
Cox proportional hazards regression models assessing the risk of mortality according to T2D status. Model 1: adjusted for age (penalized spline with 3 knots) and sex (in full model). Model 2: adjusted for: age (penalized spline with 3 knots), sex (in full model), EF category, CKD, ASCVD, hypertension, atrial fibrillation, COPD, and dyslipidemia. *ASCVD* atherosclerotic cardiovascular disease, *CI* confidence interval, *COPD* chronic obstructive pulmonary disease, *HR* hazard ratio

**Fig. 4:**
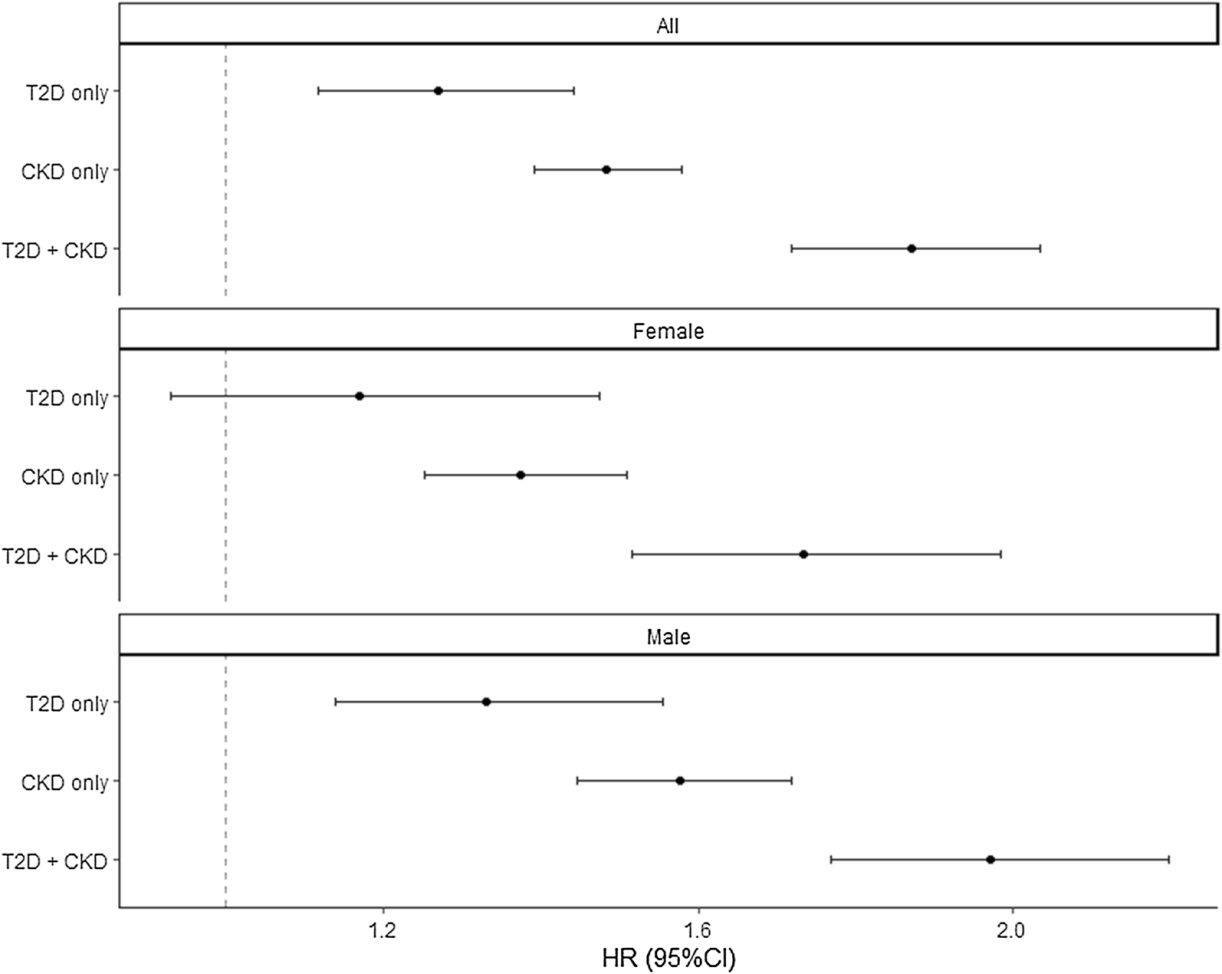
Cox proportional hazards regression models assessing the risk of mortality according to T2D and CKD status. Model adjusted for: age (penalized spline with 3 knots), sex (in full model), EF category, ASCVD, hypertension, atrial fibrillation, COPD, and dyslipidemia. *ASCVD* atherosclerotic cardiovascular disease, *CI* confidence interval, *CKD* chronic kidney disease, *COPD* chronic obstructive pulmonary disease; *EF* ejection fraction, *HR* hazard ratio, *T2D* type 2 diabetes

Figure 2 illustrates survival according to T2D status. Survival curves between diabetes groups diverged starting > 1 year after hospital admission. Cox regression models showed a higher risk of mortality among patients with T2D compared to those without (most adjusted hazard ratio, HR: 1.21, 95% CI 1.14 to 1.29), with no statistically significant difference between females (HR 1.25, 95% CI 1.13 to 1.38) and males (HR 1.19, 95% CI 1.10 to 1.29) (Fig. 3) or between HFpEF (HR 1.19, 95% CI 1.10 to 1.19) and HFrEF (HR 1.25 95% CI 1.13 to 1.37) (Additional file 1: Figure S2). When patients without T2D nor CKD were considered as a reference group, risk of mortality HR (95% CI) of those with T2D, CKD, or both, were at 1.11 (0.93 to 1.32), 1.44 (1.33 to 1.56), and 1.84 (1.66 to 2.05), respectively (Fig. 4).

**Fig. 5:**
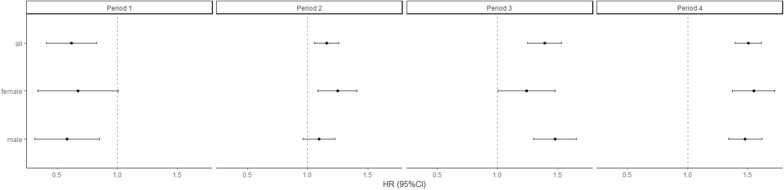
Time-partitioned Cox proportional hazards regression models assessing the risk of mortality according to T2D and CKD status, overall and stratified by sex. Model adjusted for: age (penalized spline with 3 knots), EF category, CKD, ASCVD, hypertension, atrial fibrillation, COPD, and dyslipidemia. *ASCVD* atherosclerotic cardiovascular disease, *CKD* chronic kidney disease, *COPD* chronic obstructive pulmonary disease; *HR* hazard ratio, *T2D* type 2 diabetes

Upon statistical inspection, the above models did not fulfil PH assumption, as shown by Schoenfeld’s residuals test (p  < 0.05) and illustrated on Schoenfeld residuals plot on Additional file 1: Figure S1. In the time stratified analyses, PH assumptions were met (Schoenfeld’s residuals test p > 0.05). T2D was associated with higher risk of mortality from Period 2 onwards (i.e. when observed beyond 30 days after hospitalization day 1)) (Fig. 5). The results were similar when stratified by sex (Fig. 5) or EF category (Additional file 1: Figure S3).

In the four exposure group analysis (i.e. by T2D and CKD status), T2D was not associated with an increased mortality risk in Periods 1–2 (i.e. when observed within 1 year), but was statistically significantly associated with increased risk of all-cause mortality in Periods 3–4 (when observed beyond 1 year) (Fig. 5). Meanwhile, CKD was associated with a lower risk of all-cause mortality in Period 1, but was associated with an increased risk of all-cause mortality in Periods 2–4 (Fig. 5). Similarly, those with both T2D and CKD had a lower risk of all-cause mortality in Period 1 (< 1 month), but higher risk of all-cause mortality in Periods 2–4 (Fig. 5). Moreover, in Periods 2–4, those with both T2D and CKD had higher risk of all-cause mortality than those with either T2D or CKD alone (Fig. 5).

### Survival and life expectancy

**Fig. 6:**
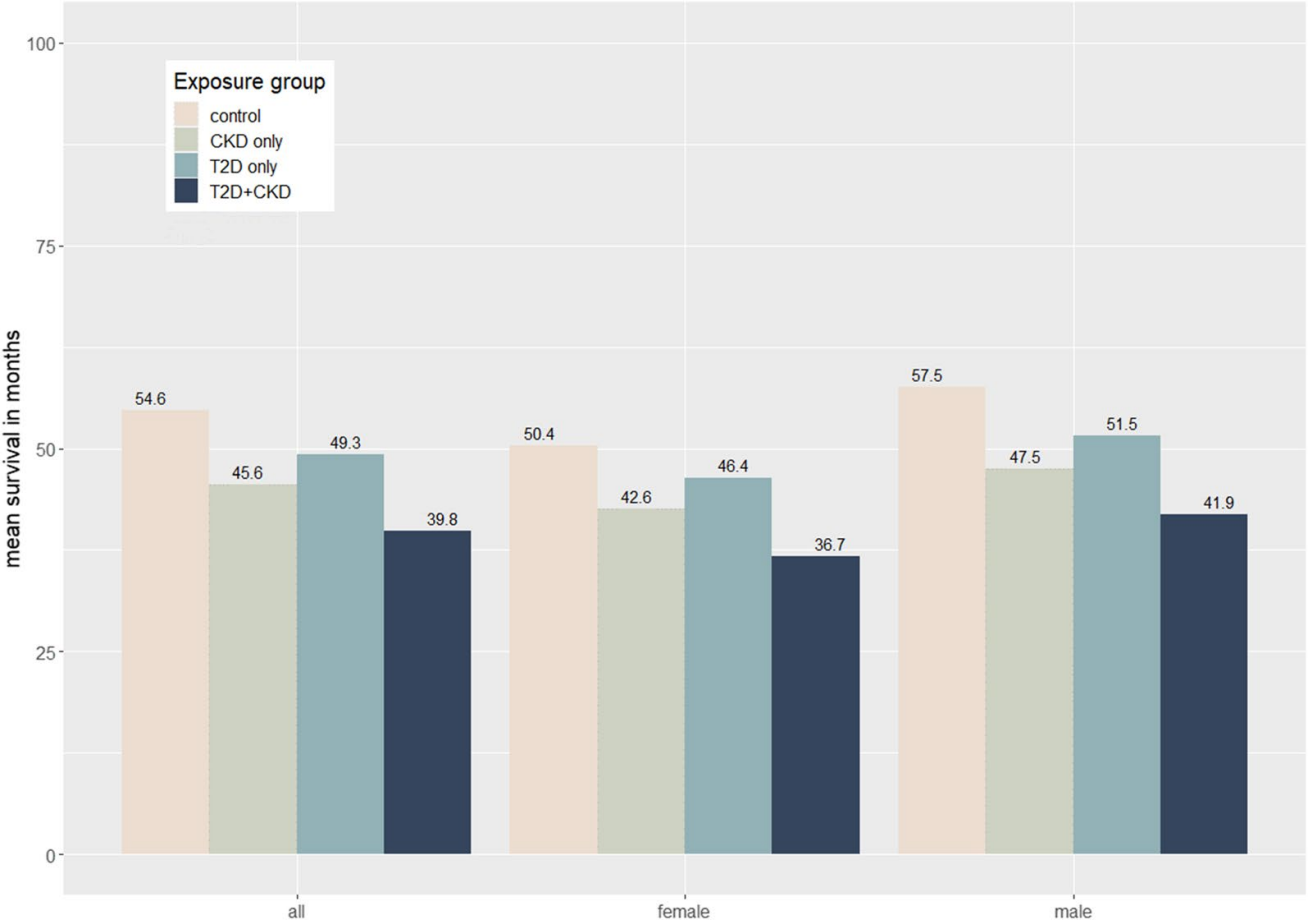
Estimated life expectancy according to T2D and CKD status, stratified by sex. *CKD* chronic kidney disease, *T2D* type 2 diabetes

Overall, patients with T2D had on average shorter life expectancy compared to those without. Furthermore, those with both T2D and CKD had even shorter life expectancy than those with T2D alone. Bootstrapped estimates showed that compared to control, average life expectancy (95% CI) among inpatients with T2D, CKD, or both was 5.4 months (1.1 to 9.7) less, 9.0 months (8.4 to 9.6) less, or 14.8 months (12.4 to 17.2) less, respectively, with no sex differences (Fig. 6).

### Sensitivity analysis

Results remained similar when after adjusting for in-hospital mortality (overall HR: 1.21, 95%CI 1.14 to 1.29) (Additional file 1: Table S2a).), and when patients that had in-hospital mortality were excluded in the analyses (Additional file 1: Table S2b).

We did not observe a statistical interaction between sex or EF category with T2D (p for interaction > 005) on its association with all-cause mortality. Stratified analyses by sex or EF category showed results were similar to overall (Fig. 3 and Additional file 1: Figure S2). In those with HFpEF, the risks of all-cause mortality were higher for each additional comorbidity (T2D, CKD, or both). In those with HFrEF, the risks of all-cause mortality were higher in those with any comorbidity (T2D, CKD, or both), with overlapping confidence intervals (Additional file 1: Figure S2).

Survival contrasts of exposure groups were similar in the HFpEF stratum: compared to control, average life expectancy (95% CI) among inpatients with T2D, CKD, or both was 2.6 months (0.3 to 4.9) less, 9.1 months (7.0 to 11.2) less, or 15.1 months (12.6, 17.6) less, respectively (Additional file 1: Figure S4). In the HFrEF stratum, average life expectancy (95% CI) among inpatients with T2D, CKD, or both was 8.8 months (5.6, 12.5) less, 9.1 months (7.0, 11.2) less, or 14.4 months (12.3, 16.4) less, respectively (Additional file 1: Figure S4).

## Discussion

In this ambispective, observational clinical and civil registry of consecutive inpatients with HF in Switzerland’s largest tertiary cardiovascular referral center, we described the country’s largest cohort inpatients with HF and analysed long-term outcomes. We observed that overall, T2D was independently associated with 21% higher risk of all-cause mortality and shorter life expectancy among inpatients with HF, even after taking into account multiple risk factors and potential confounders such age, sex, CKD, EF, ASCVD, hypertension, dyslipidemia, atrial fibrillation, and COPD. Furthermore, presence of both T2D and CKD was associated with 84% higher risk of all-cause mortality and reduced life expectancy. In patients with HFpEF, T2D confers higher risk of all-cause mortality and shorter life expectancy. CKD confers significantly higher risk than T2D, and even higher when both T2D and CKD coexist. In patients with HFrEF, the higher risks conferred by T2D or CKD were similar, but higher when both coexist.

Our study population is consistent with a large multinational study describing a contemporary population of patients with heart failure, where ASCVD such as ischemic heart disease and CKD are found to be common comorbidities [16]. Moreover, our results are in line with previous studies that have compared clinical characteristics and long-term outcomes of patients hospitalized for heart failure with or without diabetes [11, 12, 17]. In a meta-analysis of observational studies, diabetes was associated with higher risk of all-cause, long-term mortality based on pooled estimates from acute and chronic HF registries (HR 1.13, 95% CI 1.05 to 1.22, *I*^2^ = 85.3, and HR 1.44, 95% CI 1.36 to 1.52, *I*^2^ = 56.1, respectively) with longer follow-up periods up to 15 years [11]. A randomized controlled trial with 5 to 8 years of follow-up also showed diabetes as an independent risk factor for all-cause mortality [12]. Moreover, our study also is also in line with a large observational study where CKD was associated with further increases in mortality risks among patients with HF and T2D [17]. Nevertheless, our study extends previous findings by exploring the influence of CKD and T2D in HF patients, and extrapolating association estimates to life expectancy estimates.

In our analysis that stratified patients according to EF category, we did not observe a statistically significant interaction between T2D and EF category for all-cause mortality. This is in line with findings from the CHARM program, in that the magnitude of all-cause mortality did not differ between EF categories [13]. They also did not observe an interaction of T2D and EF with cardiovascular mortality as the outcome [13]. Potential interaction effects may not have been observed in our population since treatment significantly improves prognosis in HFrEF but only modestly in HFpEF,

We observed that the association of T2D on HF could be time-dependent, with similar risk of mortality in the acute term (< 30 days follow-up), but an increasing risk of mortality with longer follow-up even after accounting for age. This could partly be explained by the delayed effects of diabetes on HF, and the episode of hospitalization accelerated the longstanding maladaptive alterations, structural changes, cardiovascular dysfunction that T2D already imparts before hospitalization [18]. Moreover, our results may suggest a paradoxical benefit (i.e. HR < 1.0) of CKD in the acute term (< 30 days) in HF patients and deleterious effects afterwards. This could in part be explained by underdiagnosis of CKD in this population and hence undertreatment, whereas those who have been diagnosed early might have received appropriate treatment that may improve survival early during hospital admission. This hypothesis could be further strengthened by recent evidence showing that in general, many cases of kidney disease may be missed when using medical record data [19]. Thus, the possibility of misclassification of undiagnosed CKD with worse acute prognosis in the non-CKD group could be plausible. Investigation of these maybe warranted if acute survival is of particular interest.

Overall, we observed that T2D and CKD were associated with higher risk of mortality among HF inpatients in the long term (i.e. beyond acute period). We extended evidence by translating relative risks into absolute measures more interpretable to the patients, clinicians, and policymakers. Inpatients with heart failure and T2D had shorter survival than those without T2D, those with CKD had shorter survival than those with T2D whilst those with both T2D and CKD had shorter survival than those with either T2D or CKD alone. These estimates constitute a substantial life expectancy reduction, considering that our patient population is relatively older and represents patient populations that already have a very high mortality risk. Moreover, healthcare costs at this age and disease severity are expected to increase, and particularly among patients with CKD who incur much higher hospital care costs, this could mean even much greater societal costs [16].

The observed association of diabetes on long-term survival among HF inpatients could be explained by ischemic and non-ischemic mechanisms [1, 18]: insulin resistance and hyperglycemia worsen dyslipidemia, accelerate atherosclerosis, and induce inflammation, promoting leucocyte adhesion and coronary plaque formation, eventually leading to plaque rupture and coronary thrombosis [1]. Hyperglycemia activates renin–angiotensin–aldosterone system (RAAS) and cytokines, which cause endothelial dysfunction and vasospasm [18]. Diabetes also increases transforming growth factor-β (TGF-β) directly through gene upregulation or indirectly through RAAS activation, which in turn increase TGF-β [20]. TGF-β triggers a cascade leading to formation of cardiac fibrosis, leading to structural changes and dysfunction [21]. In a meta-analysis, diabetes was associated with higher degrees of cardiovascular magnetic resonance imaging-derived estimates myocardial fibrosis [22], and higher degrees of myocardial fibrosis have been associated with all-cause mortality [23]. In patients with CKD, kidneys can release hormones and inflammatory cytokines that influence vascular tone, and hemodynamic alterations further affect the failing heart [24]. Moreover, heart failure induced renal hypoperfusion activates RAAS, sympathetic nervous system, and arginine vasopressin that leads to fluid retention, increased preload, and worsening heart failure [25].

To our knowledge, this is the first study that reported relative (i.e. HR) and absolute estimates (i.e. months survival) on the association of T2D with life expectancy among inpatients with HF, increasing bedside interpretability. We posit that after hospital admission with advanced stages of disease, life expectancy is a more useful information that could improve risk communication from clinicans to patients. Another strength of our study relies on its large, real - world hospital setting of inpatients with HF patients in Switzerland, exceeding the size of some multinational registries of HF in Europe [26–28]. This large sample size provided us with sufficient power even when time-stratified and sensitivity analyses were performed. Through the hospital’s data science center, we were able to obtain information on mortality events over a relatively long period, thus we were able to estimate long-term mortality and robust life expectancy estimates. Because we included all inpatients with HF whether or not HF was the primary reason for admission, our results are thus more generalizable to the inpatient population. We were able to account for the relative impact of CKD, on the association between T2D and mortality, instead of only adjusting or stratifying as most studies have done.

Our findings should be interpreted against several considerable limitations. First, the data we analysed were based on medical records at the hospital. Thus, we lacked information on routine subclinical measures that stratify risk in patients with T2D or CKD: glycosylated hemoglobin and estimated glomerular filtration rate, that could have provided important insights clinical risk stratification. Further, data on cause of death were not available, which precluded us from exploring whether our findings were driven by CV deaths. This could have allowed us to compare results with other similar studies. Second, our study lacked information on durations of T2D, HF, and other comorbidities such as CKD. Our reliance on ICD-10 codes to identify most T2D and CKD cases could also have led to misclassification of non-T2D and CKD cases if the ICD code was not encoded, hence in the non-T2D group some cases of T2D maybe present. This is a pattern that is in general observed when using medical record data [19]. However, if a negative association between T2D and mortality exists, that would have led to an underestimation of the association in our study, especially in long-term. Third, in our sensitivity analyses, we excluded patients who died during the same episode of hospital admission, and this may have introduced selection bias. However, mortality rates between those included and excluded were comparable. Fourth, we were not able to account for smoking, alcohol intake, and other lifestyle factors. However, we were able to account for most risk factors for mortality and confounders in the association between diabetes and mortality among patients with HF. Many patients lacked BMI measurements; however, mortality rates did not differ between groups in those with or without BMI measurements, suggesting low likelihood for selection bias to have occurred. Fifth, since our study involved high risk patients in Switzerland, these findings may only be generalized to patients with advanced risk profiles and those in similar healthcare settings. Finally, in the time stratified analyses, group sizes among females were small hence underpowered, and could likely explain the reason why no significant differences in risks were found between diabetes groups across all periods.

In conclusion, we showed that T2D is associated with a significantly higher risk of all-cause mortality with significant reduction in life expectancy among patients with HF, even when clinical risk factors and potential confounders such as age, sex, hypertension, EF, CKD, ASCVD, and other comorbidities were taken into account. Among those with T2D, CKD was associated with further reduction in life expectancy.

## Supplementary Information

**Additional file 1: Table S1**. Baseline characteristics of patients hospitalised for heart failure at Inselspital, 2015-2019, by sex. **Figure S1**. Schoenfeld residuals plotted against time. **Table S2a**. Time-partitioned Cox proportional hazards regression models* assessing the risk of mortality according to diabetes status, adjusting for in-hospital mortality. **Table S2b**. Time-partitioned Cox proportional hazards regression models* assessing the risk of mortality according to diabetes status, excluding patients that had in-hospital mortality. **Figure S2**. Cox proportional hazards regression models* assessing the risk of mortality according to T2D status, stratified by ejection fraction. **Figure S3**. Time-partitioned Cox proportional hazards regression models* assessing the risk of mortality according to T2D status, stratified by ejection fraction. **Figure S4**. Estimated life expectancy according to diabetes and CKD status, stratified by EF.

## Data Availability

The datasets used and/or analysed during the current study are available from the corresponding author on reasonable request and upon approval of the relevant institutional review boards.

## Abbreviation

ASCVD: Atherosclerotic cardiovascular disease
ATC: Anatomical Therapeutic Chemical Classification System
CKD: Chronic kidney disease
COPD: Chronic obstructive pulmonary disease
EF: Ejection fraction
HF: Heart failure
HR: Hazard ratio
ICD-10: The International Statistical Classification of Diseases and Related Health Problems 10th version
RAAS: Renin angiotensin aldosterone system
T2D: Type 2 diabetes
