# Supplementary material for: Impact of type 2 diabetes on life expectancy and role of kidney disease among inpatients with heart failure in Switzerland: an ambispective cohort study
Source: Cardiovasc Diabetol. 2023 Jul 12;22:174. doi: 10.1186/s12933-023-01903-7 (PMC10339473; doi:10.1186/s12933-023-01903-7)
Supplement: Supplementary file 1 — Additional file 1: Table S1. Baseline characteristics of patients hospitalised for heart failure at Inselspital, 2015-2019, by sex. Figure S1. Schoenfeld residuals plotted against time. Table S2a. Time-partitioned Cox proportional hazards regression models* assessing the risk of mortality according to diabetes status, adjusting for in-hospital mortality. Table S2b. Time-partitioned Cox proportional hazards regression models* assessing the risk of mortality according to diabetes status, excluding patients that had in-hospital mortality. Figure S2. Cox proportional hazards regression models* assessing the risk of mortality according to T2D status, stratified by ejection fraction. Figure S3. Time-partitioned Cox proportional hazards regression models* assessing the risk of mortality according to T2D status, stratified by ejection fraction. Figure S4. Estimated life expectancy according to diabetes and CKD status, stratified by EF. [file 12933_2023_1903_MOESM1_ESM.docx]

**Additional file Table S1. Baseline characteristics of patients hospitalised for heart failure at Inselspital, 2015-2019, by sex**

|  | Female | Male |
| --- | --- | --- |
| n (%) | 4,218 (40%) | 6,314 (60%) |
| Age, median [IQR] | 81.0 [72.5, 87.1] | 74.1 [65.0, 82.1] |
| BMI, median [IQR] | 25.4 [22.0, 29.6] | 26.4 [23.6, 30.0] |
| *Medical history* |  |  |
| Type 2 diabetes | 958 (23%) | 1,846 (29%) |
| Chronic kidney disease | 1,873 (44%) | 2,529 (40%) |
| ASCVD | 1,358 (32%) | 3,092 (49%) |
| Hypertension | 2,396 (57%) | 3,401 (54%) |
| Atrial fibrillation | 1,353 (32%) | 2,006 (32%) |
| Dyslipidaemia | 69 (2%) | 145 (2%) |
| Chronic obstructive pulmonary disease (COPD) | 333 (8%) | 299 (5%) |
| *Medications* |  |  |
| Insulin | 760 (18%) | 1,503 (24%) |
| Oral antidiabetic medications | 491 (12%) | 976 (16%) |
| SGLT2 inhibitors | 41 (1%) | 141 (2%) |
| GLP1 receptor analogs | 29 (1%) | 80 (1%) |
| Antithrombotic medications | 3,810 (90%) | 5,932 (94%) |
| Digoxins and nitrates | 1,267 (30%) | 1,633 (26%) |
| Diuretics | 3,496 (83%) | 5,237 (83%) |
| Beta-blockers | 3,068 (73%) | 5,067 (80%) |
| RAAS inhibitors | 3,139 (74%) | 5,225 (83%) |
| Ejection fraction, % median [IQR] | 45 [30, 60] | 45 [35, 60] |
| HFpEF % | 2442 (58%) | 3706 (59%) |
| HFrEF % | 1776 (42%) | 2608 (41%) |
| *Deaths* | 2,331 (55%) | 3,016 (48%) |

*Abbreviations:* ASCVD – atherosclerotic cardiovascular disease; BMI – body mass index; COPD – chronic obstructive pulmonary disease; GLP-1 – glucagon-like peptide-1; RAAS – renin-angiotensin-aldosterone system; SGLT2 – sodium-glucose cotransporter 2

**Additional file Figure S1. Schoenfeld residuals plotted against time**

**
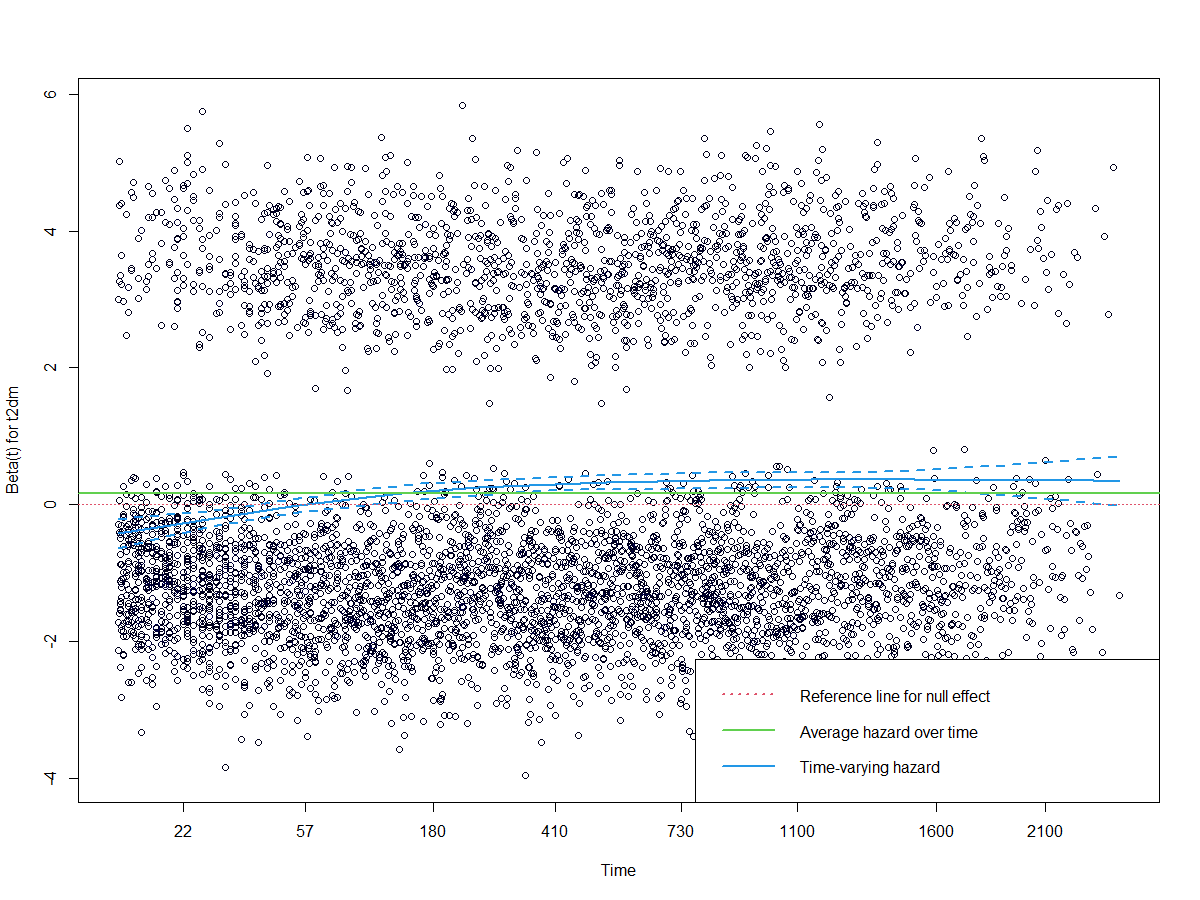
**

Schoenfeld residuals (dots) were plotted against time in days. The hazard associated with diabetes shows time variation, where it starts negative, traverses 0 (null effect) at about 60 days, then stays positive and stable beyond 1 year.

**Additional file Table S2a. Time-partitioned Cox proportional hazards regression models* assessing the risk of mortality according to diabetes status, adjusting for in-hospital mortality**

| Time partition | Number of participants | Number of events | HR (95% CI)* | Schoenfeld’s residual test (p) |
| --- | --- | --- | --- | --- |
| <1 month | 10532 | 730 | **0.62 (0.51, 0.77)** | <0.01 |
| Female | 4218 | 321 | **0.69 (0.49, 0.97)** | <0.01 |
| Male | 6314 | 409 | **0.57 (0.44, 0.75)** | <0.01 |
| 1 month to 1 year | 9802 | 1979 | **1.14 (1.03, 1.27)** | 0.07 |
| Female | 3897 | 870 | **1.25 (1.06, 1.47)** | 0.06 |
| Male | 5905 | 1109 | 1.07 (0.94, 1.23) | 0.07 |
| 1 year to 2 years | 7823 | 963 | **1.39 (1.21, 1.60)** | 0.87 |
| Female | 3027 | 402 | 1.23 (0.97, 1.56) | 0.78 |
| Male | 4796 | 561 | **1.48 (1.24, 1.77)** | 0.94 |
| >2 years | 6860 | 1675 | **1.50 (1.35, 1.68)** | 0.98 |
| Female | 2625 | 738 | **1.55 (1.30, 1.84)** | 0.84 |
| Male | 4253 | 937 | **1.48 (1.29, 1.70)** | 0.96 |

Adjusted for: age (penalized spline with 3 knots), CKD, ASCVD, hypertension, atrial fibrillation, COPD, and dyslipidemia

*Abbreviations:* ASCVD – atherosclerotic cardiovascular disease; CI – confidence interval; CKD – chronic kidney disease; COPD – chronic obstructive pulmonary disease; HR – hazard ratio

**Additional file Table S2b. Time-partitioned Cox proportional hazards regression models* assessing the risk of mortality according to diabetes status, excluding patients that had in-hospital mortality**

| Time partition | number of participants | Number of events | HR (95% CI)* | Schoenfeld’s residual test (p) |
| --- | --- | --- | --- | --- |
| <1 month | 10328 | 539 | **0.58 (0.45, 0.74)** | 0.71 |
| Female | 4213 | 228 | 0.71 (0.48, 1.06) | 0.49 |
| Male | 6205 | 311 | **0.50 (0.37, 0.69)** | 0.63 |
| 1 month to 1 year | 9789 | 1966 | **1.16 (1.04, 1.28)** | 0.09 |
| Female | 3895 | 868 | **1.25 (1.06, 1.47)** | 0.16 |
| Male | 5894 | 1098 | 1.10 (0.96, 1.25) | 0.07 |
| 1 year to 2 years | 7823 | 963 | **1.39 (1.21, 1.60)** | 0.87 |
| Female | 3027 | 402 | 1.23 (0.97, 1.56) | 0.78 |
| Male | 4796 | 561 | **1.48 (1.24, 1.77)** | 0.94 |
| >2 years | 6860 | 1675 | **1.50 (1.35, 1.68)** | 0.98 |
| Female | 2625 | 738 | **1.55 (1.30, 1.84)** | 0.84 |
| Male | 4253 | 937 | **1.48 (1.29, 1.70)** | 0.96 |

Adjusted for: age (penalized spline with 3 knots), CKD, ASCVD, hypertension, atrial fibrillation, COPD, and dyslipidemia

*Abbreviations:* ASCVD – atherosclerotic cardiovascular disease; CI – confidence interval; CKD – chronic kidney disease; COPD – chronic obstructive pulmonary disease; HR – hazard ratio

**Additional file Figure S2. Cox proportional hazards regression models* assessing the risk of mortality according to T2D status, stratified by ejection fraction**

**
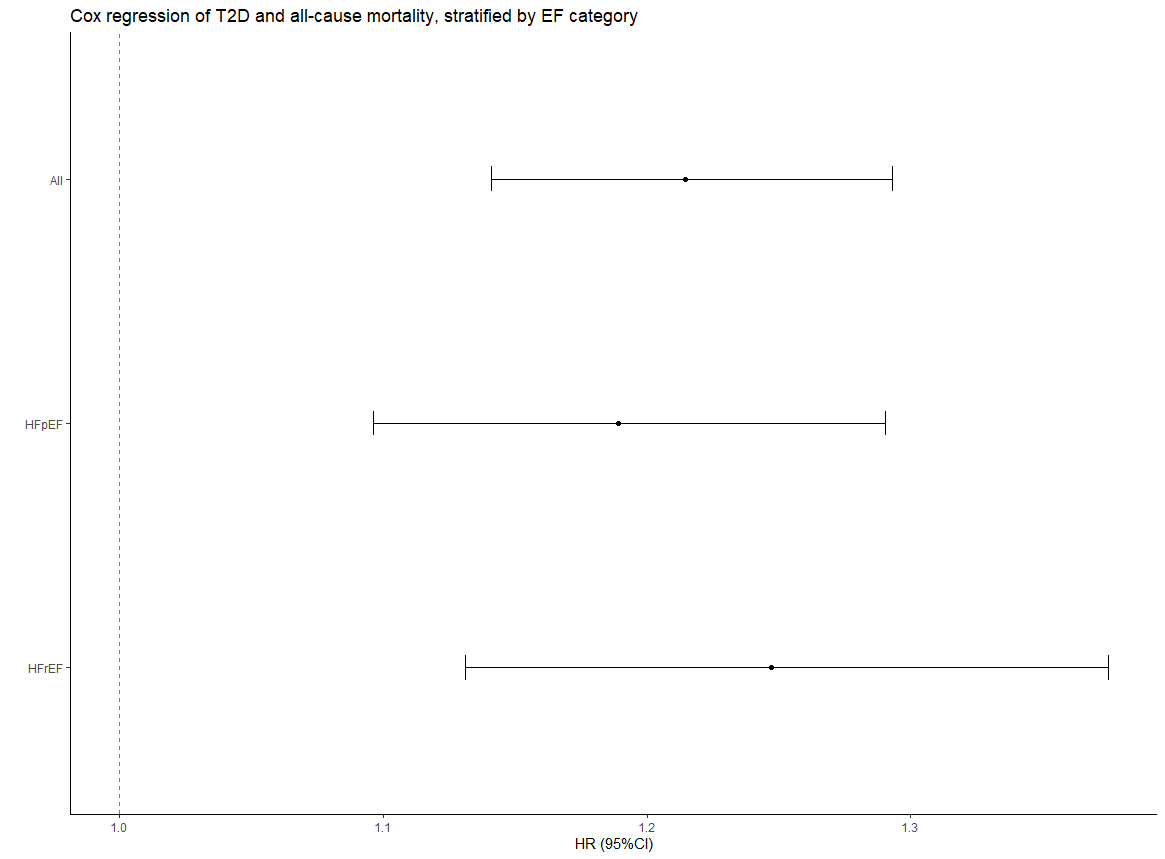
**

* adjusted for: age (penalized spline with 3 knots), sex, CKD, ASCVD, hypertension, atrial fibrillation, COPD, and dyslipidemia

*Abbreviations:* ASCVD – atherosclerotic cardiovascular disease; CI – confidence interval; CKD – chronic kidney disease; COPD – chronic obstructive pulmonary disease; EF – ejection fraction; HR – hazard ratio; T2D – type 2 diabetes

**Additional file Figure S3. Time-partitioned Cox proportional hazards regression models* assessing the risk of mortality according to T2D status, stratified by ejection fraction**

**
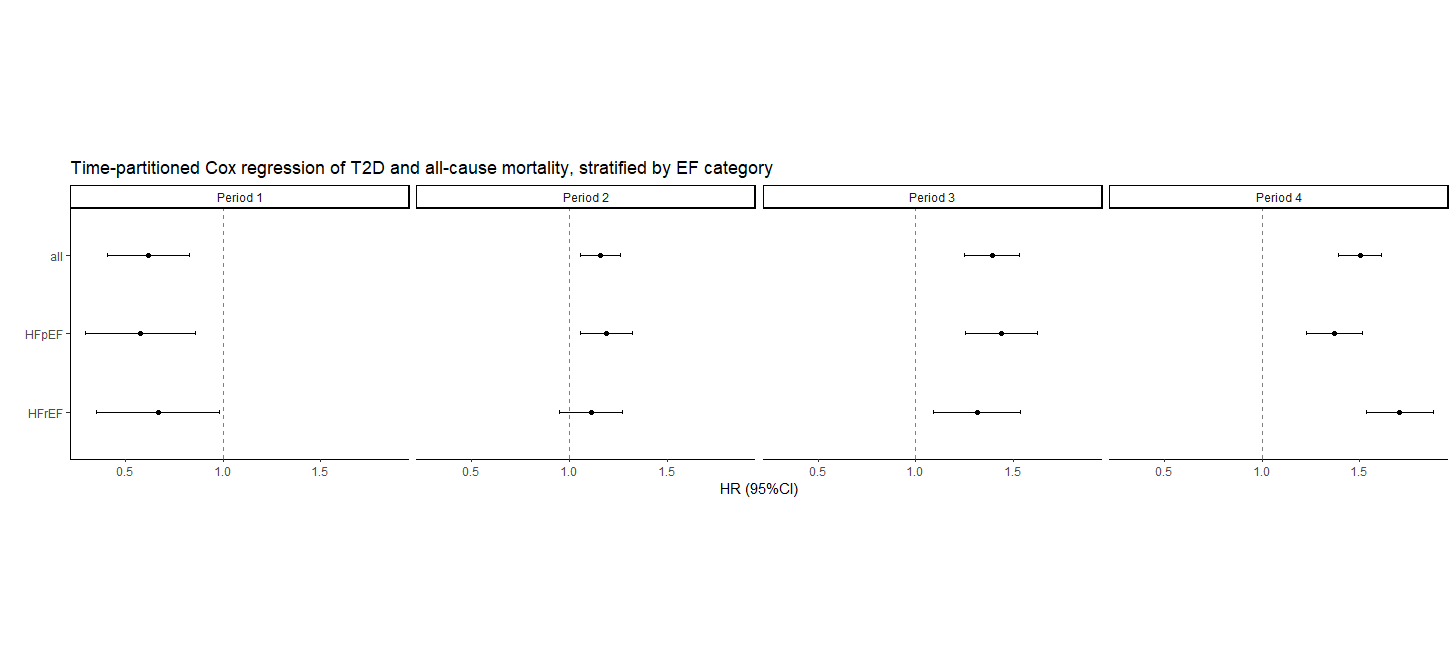
**

* adjusted for: age (penalized spline with 3 knots), sex, CKD, ASCVD, hypertension, atrial fibrillation, COPD, and dyslipidemia

Abbreviations: ASCVD – atherosclerotic cardiovascular disease; CKD – chronic kidney disease; COPD – chronic obstructive pulmonary disease; HR – hazard ratio; T2D – type 2 diabetes

**Additional file Figure S4. Estimated life expectancy according to diabetes and CKD status, stratified by EF category**
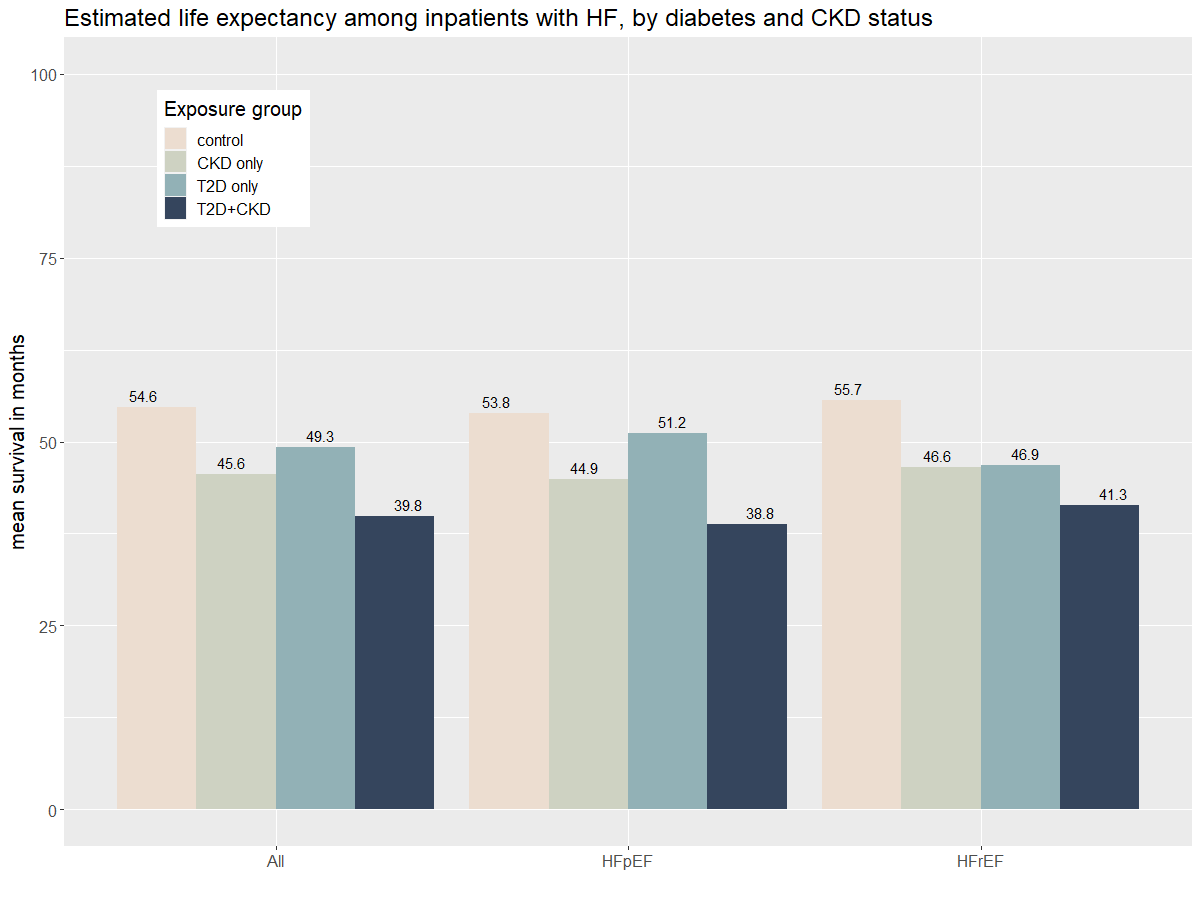


*Abbreviations:* CKD – chronic kidney disease; T2D – type 2 diabetes
